# Comparative Outcomes of Fine Needle Aspiration and Core Needle Biopsy for Parotid Masses

**DOI:** 10.1002/ohn.70305

**Published:** 2026-06-03

**Authors:** Lisa Eleni Chionis Buhler, Andrew Prince, Nicholas Bunda, Kimberly Oslin, Joshua Smith, Michael Allevato, Emma Hershey, Steven B. Chinn, Mark E. P. Prince

**Affiliations:** ^1^ Department of Otolaryngology–Head and Neck Surgery University of Michigan Ann Arbor Michigan USA; ^2^ Department of Medicine and Life Sciences University of Toledo Toledo Ohio USA; ^3^ Present address: Department of Otolaryngology–Head and Neck Surgery Kaiser Permanente Oakland California USA; ^4^ Present address: Department of Otolaryngology–Head and Neck Surgery Henry Ford Health Detroit Michigan USA; ^5^ Present address: Department of Otolaryngology–Head & Neck Surgery University of Pittsburgh Pittsburgh Pennsylvania USA; ^6^ Present address: Section of Otolaryngology–Head & Neck Surgery Dartmouth Hitchcock Medical Center Lebanon New Hampshire USA

**Keywords:** core needle biopsy, diagnostic accuracy, fine‐needle aspiration, parotid tumor, salivary gland

## Abstract

**Objective:**

To compare the diagnostic accuracy and safety of ultrasound‐guided fine needle aspiration (FNA) versus core needle biopsy (CNB) for parotid masses.

**Study Design:**

Retrospective cohort study.

**Setting:**

Tertiary academic medical center, 2018 to 2023.

**Methods:**

We reviewed 485 patients undergoing ultrasound‐guided parotid biopsy (426 FNAs and 59 CNBs). Permanent histopathology served as the reference standard. Primary outcomes included diagnostic performance metrics for neoplasm, malignancy, and tissue‐specific diagnosis. Secondary outcomes included nondiagnostic rates, complications, time to surgery, and technical factors (needle gauge, biopsy setting, and number of passes).

**Results:**

CNB demonstrated superior sensitivity (80.0% vs 54.9%, *P* = .032), accuracy (86.4% vs 68.5%, *P* = .007), and positive predictive value (87.0% vs 56.4%, *P* = .01) for detecting malignancy compared to FNA. CNB had superior tissue‐specific accuracy (67.8% vs 49.5%, *P* = .009), particularly for squamous cell carcinoma and mucoepidermoid carcinoma. FNA had a higher nondiagnostic rate (20.0% vs 5.1%, *P* = .004). Greater CNB accuracy correlated with ≥3 needle passes and use of 17 to 18 gauge needles. Complication rates were low and similar (3.4% CNB vs 2.1% FNA), with no facial nerve injuries or tumor seeding reported. Time to surgery was comparable (CNB 51 days vs FNA 42 days, *P* = .10) in cases of malignancy.

**Conclusion:**

CNB showed improved diagnostic accuracy and fewer nondiagnostic results than FNA for parotid masses, particularly for suspected malignancies and complex histologies, without added risk or delay. CNB should be considered in cases suspicious for malignancy or where tissue‐specific diagnosis would inform operative planning.

Accurate and timely preoperative diagnosis of parotid masses is essential for surgical planning, patient counseling, and avoiding unnecessary procedures. However, diagnosing parotid lesions is complex due to the heterogeneity of salivary gland tumors, which encompasses more than 18 benign and 21 malignant subtypes, each with unique prognostic and management implications.^1^, ^2^

Fine needle aspiration (FNA) is widely used because it is safe, well‐tolerated, and inexpensive, but its diagnostic performance varies, largely due to specimen adequacy, operator technique, and cytopathologist interpretation.^3^, ^4^ Core needle biopsy (CNB) is an alternative offering improved diagnostic yield and histologic specificity, reducing the need for repeat sampling.^3^, ^5^ Despite this, CNB adoption has been limited by concerns regarding invasiveness, complications, and delays in diagnosis.^3^

While several studies have compared the diagnostic performance of FNA and CNB, few have comprehensively evaluated outcomes such as nondiagnostic rates, repeat biopsy frequency, biopsy‐related complications, and surgical delays. Few studies differentiate between benign and malignant findings or include nondiagnostic specimens in diagnostic performance calculations, which may overestimate performance.^6^ Furthermore, technical factors such as biopsy location, needle gauge, and number of passes remain underexplored in the literature for parotid masses.

Our primary aim was to compare the diagnostic performance of ultrasound‐guided FNA and CNB for detecting neoplasm and malignancy in patients who ultimately underwent parotidectomy, using final surgical histopathology as the reference standard. We hypothesized no difference in diagnostic accuracy between modalities. We performed univariate analyses to evaluate associations between biopsy‐, lesion‐, and operator‐level factors and diagnostic accuracy. Secondarily, we evaluated underexplored factors including nondiagnostic rates, rapid on‐site evaluation (ROSE) utilization, complications, repeat biopsy rates, surgical delays, and the influence of procedural characteristics on diagnostic performance. Findings will help establish greater evidence for selecting the optimal parotid biopsy technique.

## Methods

This single‐center retrospective cohort study was conducted at a tertiary academic center and received Institutional Review Board exemption (HUM00223009). We included patients ≥18 years who underwent parotidectomy with facial nerve (CN7) preservation between 2018 and 2023. Patients without preoperative biopsy, intraoperative frozen section biopsies only, biopsies not performed with ultrasound, excisional lymph node biopsies, biopsies outside the parotid, and superficial shaves or punches that did not involve glandular tissue were excluded. This resulted in 485 patients for analysis and 531 total ultrasound‐guided biopsies if multiple were included. Revision parotidectomies (5.8%) and metastatic lesions infiltrating the parotid were retained to reflect real‐world practice.

Demographic and comorbidity variables included age, sex, race or ethnicity, body mass index (BMI), Charlson Comorbidity Index (CCI), anticoagulant/antiplatelet therapy, bleeding diathesis, and tobacco/alcohol use. Biopsy characteristics included modality (FNA or CNB), biopsy setting (home vs outside institution), operator specialty and training level, needle gauge, number of passes/samples obtained, and complications. For home institution FNAs, ROSE utilization was recorded. Pathology data included the diagnostic impression, which was scored according to the Milan System^7^; Milan subcategories 4A and 4B were combined because few reports distinguished them. For analyses assessing diagnostic performance for malignancy, Milan categories 5 and 6 were considered positive for malignancy. Categories 1 to 4 were considered negative. Biopsies categorized as AUS or SUMP with malignant surgical pathology were classified as false negatives. For outside‐institution biopsies that underwent pathology transfer consultation at the home institution, the overread diagnosis was used instead of the external results for standardization.

Primary outcomes included diagnostic performance of FNA and CNB for detecting neoplasia, malignancy, and tissue‐specific diagnoses (ie, the accuracy of the biopsy in identifying the exact pathologic diagnosis). Secondary endpoints included nondiagnostic rates, repeat biopsy frequency, biopsy complications, procedural factors influencing accuracy, unnecessary surgeries (ie, performed following concerning or nondiagnostic preoperative biopsy but pathology demonstrated normal tissue, scar, inflammation, or fibrosis), and timing of surgery. Days from biopsy to surgery were examined to assess whether CNB, often scheduled through outside specialties and requiring additional referral time, had a similar timing of surgery compared with point‐of‐care FNA.

Each biopsy was cross‐tabulated against permanent postoperative histopathology to obtain true‐positive, true‐negative, false‐positive, and false‐negative counts. Sensitivity, specificity, positive and negative predictive values, accuracy, and tissue‐specific diagnostic accuracy were calculated. We included and excluded inconclusive results in separate analyses. For patients who underwent multiple biopsies, only the first biopsy in the sequence was included in the diagnostic performance analysis to avoid duplication and bias.

Continuous variables were compared using an unpaired *t* test, and categoric variables were expressed by using counts or percentages, and differences between FNA and CNB were tested with Fisher's exact and two‐sided chi‐squared tests (when counts were ≥10). McNemar testing was used to compare results from the same cohort. Missing values were excluded. Analyses were performed in SPSS 26. Statistical significance was defined as *P* < .05.

## Results

There were 485 participants in the final analytic cohort. Demographics were balanced between modalities (Table 1). There were no differences in CCI, anticoagulant or antiplatelet use, tobacco and alcohol exposure, prior parotid surgery, or prior radiation between groups. Mean tumor size was 2.7 ± 1.5 cm, similar between FNA and CNB cohorts.

**Table 1:** Patient Characteristics

Variable	Total (N = 485 [%])	FNA (N = 426 [%])	CNB (N = 59 [%])	Statistic
*Sex*				.785
Male	255 (52.6)	223 (52.3)	32 (54.2)	
Female	230 (47.4)	203 (47.7)	27 (45.8)	
*Alcohol use, n (%)*				.573
Never	282 (58.1)	245 (57.5)	37 (62.7)	
Past	123 (25.4)	108 (25.4)	15 (25.4)	
Current	80 (16.5)	73 (17.1)	7 (11.9)	
*Anticoagulation use*	42 (8.7)	40 (9.4)	2 (3.4)	.144
*Aspirin use*	80 (16.5)	72 (16.9)	8 (13.6)	.584
*Bleeding diathesis*	6 (1.2)	5 (1.2)	1 (1.7)	.546
*Recreational or illicit drug use*				.362
Never	453 (93.4)	396 (93.0)	57 (96.6)	
Past	14 (2.9)	14 (3.3)	0 (0.0)	
Current	18 (3.7)	16 (3.8)	2 (3.4)	
*Ethnicity*				.434
Hispanic or Latino	13 (2.7)	13 (3.1)	0 (0.0)	
Non‐Hispanic or Latino	456 (94.0)	399 (93.7)	57 (96.6)	
Unknown	8 (1.6)	7 (1.6)	1 (1.7)	
Patient refused	5 (1.0)	5 (1.2)	0 (0.0)	
*Prior parotid surgery*	28 (5.8)	24 (5.6)	4 (6.8)	.764
*Prior radiation to parotid*	8 (1.6)	8 (1.9)	0 (0.0)	.604
*Race*				.517
White	411 (84.7)	363 (85.2)	48 (81.4)	
African American	28 (5.8)	21 (4.9)	7 (11.9)	
Asian	15 (3.1)	14 (3.3)	1 (1.7)	
Other	15 (3.1)	14 (3.3)	1 (1.7)	
Patient refused	3 (0.6)	2 (0.5)	1 (1.7)	
Unknown	2 (0.4)	3 (0.7)	0 (0.0)	
*Tobacco use*				.152
Never	243 (50.1)	217 (50.9)	26 (44.1)	
Past	138 (28.5)	124 (29.1)	14 (23.7)	
Current	103 (21.2)	85 (20.0)	18 (30.5)	
*Charlson Comorbidity Index*				.524
0 to 2	224 (46.2)	196 (46.0)	28 (47.5)	
3 to 5	147 (30.3)	129 (30.3)	18 (30.5)	
6 to 8	70 (14.4)	59 (13.8)	11 (18.6)	
9 to 11	34 (7.0)	32 (7.5)	2 (3.4)	
>12	10 (2.1)	10 (2.3)	0 (0.0)	
	**Mean ± SD**			
*BMI, kg/m^2^*	30.17 ± 7.35	30.22 ± 7.37	29.86 ± 7.24	.727
*Age at biopsy, y*	59.77 ± 16.36	59.57 ± 16.55	61.22 ± 14.98	.467
*Tumor size, cm, greatest dimension*	2.72 ± 1.50	2.70 ± 1.39	2.85 ± 2.12	.505

Abbreviations: BMI, body mass index; CNB, core needle biopsy; FNA, fine needle aspiration.

Across 485 participants, 577 biopsies (490 FNAs, 87 CNBs) were performed. There were 58 patients (12.0%) with >1 biopsy of the same lesion. Most patients (77.7%) underwent a single FNA, 10.3% underwent a single CNB, repeat biopsies were required in 47 patients (85% after initial FNA), and the remainder underwent paired procedures in various sequences (Table 2).

**Table 2:** Biopsy Sequences^a^

Biopsy sequence	Frequency	Percent of total patients (N = 485), %
FNA only	377	77.73
CNB only	50	10.31
FNA then repeat FNA	26	5.36
FNA then CNB	11	2.27
Concurrent FNA and CNB	11	2.27
CNB then FNA	6	1.24
FNA then repeat FNA with concurrent CNB	3	0.62
Concurrent FNA and CNB then repeat FNA	1	0.21
Total	485	100.00

Abbreviations: CNB, core needle biopsy; FNA, fine needle aspiration.

^a^ Excluded any shave, punch, excisional, or other biopsies that may have occurred during this sequence.

The difference in biopsy setting between FNA and CNB was significant (*P* < .001) with CNBs performed more often at outside institutions (74.6%) compared to the home institution (Table 3). Operator specialty differed significantly (*P* < .001): FNA was performed most frequently by otolaryngologists (53.5%), followed by interventional radiologists (14.8%) and diagnostic radiologists (11.0%), whereas CNB was predominantly performed by diagnostic (40.7%) and interventional radiologists (39.0%), with 6.8% performed by otolaryngologists. Most outside‐institution biopsies were conducted by non‐otolaryngology specialties (n = 176, 76.9%), most commonly radiology (n = 98, 42.8%); in 66 cases (28.8%), the specialty was not reported. Biopsy modality varied by specialty: outside radiologists predominantly performed CNB (n = 33, 75%), whereas outside otolaryngologists rarely used CNB (n = 2, 4.5%) (*P* < .001). Home institution biopsies were primarily performed by otolaryngology (n = 179, 69.9%), who overwhelmingly used FNA (n = 177, 98.9%). Home radiologists performed CNB in 22.0% (n = 12) of biopsies, compared with otolaryngologists performing CNB in 1.1% (n = 2) of biopsies (*P* < .001).

**Table 3:** Biopsy Characteristics

	FNA		CNB		
	N = 426	%	N = 59	%	Statistic
*Biopsy setting*					<.001*
University of Michigan	241	56.57	15	25.42	
Outside hospital	185	43.43	44	74.58	
*Operator specialty*					<.001*
Otolaryngology	228	53.52	4	6.78	
Interventional radiology	63	14.79	23	38.98	
Diagnostic radiology	47	11.03	24	40.68	
Pathology	26	6.10	0	0.00	
General surgery	1	0.23	0	0.00	
Endocrinology	1	0.23	0	0.00	
Emergency medicine	0	0.00	1	1.69	
Oral/maxillofacial surgery	1	0.23	0	0.00	
Not reported	59	13.85	7	11.86	
*Operator training level*					.316
Resident	52	12.21	4	6.78	
Attending	255	59.86	41	69.49	
Physician assistant	40	9.39	7	11.86	
Fellow	19	4.46	0	0.00	
Registered diagnostic medical sonographer	59	13.85	7	11.86	
Not reported	1	0.23	0	0.00	
*Milan criteria*					.073
Nondiagnostic	43	10.09	2	3.39	
Non‐neoplastic	42	9.86	5	8.47	
Atypia of undetermined significance	34	7.98	1	1.69	
Neoplasm	212	49.77	30	50.85	
Suspicious for malignancy	2	0.47	0	0.00	
Malignant	92	21.60	21	35.59	
Not reported	1	0.23	0	0.00	
*Complications*					.639
Total yes	9	2.11	2	3.39	
Total no	346	81.22	50	84.75	
Not reported	71	16.67	7	11.86	
*Complication categories*					
Pain, bruising, swelling	4	0.82	1	1.15	
Bleeding	2	0.41	0	0.00	
Superficial infection	1	0.20	0	0.00	
Biopsy aborted due to pain	1	0.20	0	0.00	
“Negative experience”	1	0.20	0	0.00	
“Couldn't obtain, switched to CNB”	0	0.00	1	1.15	

Abbreviations: CNB, core needle biopsy; FNA, fine needle aspiration.

Turnaround time for pathology results was similar between CNB (2.8 ± 2.3 days) and FNA (2.4 ± 2.3 days) (Table 4). For cases with malignant surgical pathology, the median time from initial biopsy to surgery was 42 days for FNA and 51 days for CNB (*P* = .10) (Table 4). Complications were low and comparable between groups (3.4% CNB vs 2.1% FNA, *P* = .64), consisting mainly of transient pain or bruising. No CN7 injuries, tumor seeding, or clinically significant bleeding occurred.

**Table 4:** Additional Biopsy Characteristics

	FNA		CNB		
	Mean	SD	Mean	SD	Statistic
Needle gauge size	23.07	1.69	18.54	1.37	<.001*
Number of passes/samples	3.80	1.32	3.57	1.45	.292
Days until results	2.36	2.26	2.79	2.32	.174
Days until surgery	250.34	587.45	170.86	228.01	.305
Days until surgery (malignant surgical pathology only)	105.13	255.28	113.60	187.10	.84

	Median	IQR	Median	IQR	Statistic
Days until surgery	56.00	33.5‐133.5	70.00	42.0‐116.0	.189
Days until surgery (malignant surgical pathology only)	42.00	29.00‐66.50	51.00	29.00‐86.00	.10

Abbreviations: CNB, core needle biopsy; FNA, fine needle aspiration; IQR, interquartile range.

With all biopsies included, there was no difference between FNA and CNB for detecting neoplasia across sensitivity, specificity, positive predictive value (PPV), negative predictive value, or overall accuracy (Table 5). For malignancy, CNB outperformed FNA across all metrics, with greater sensitivity (80.0% vs 54.9%, *P* = .032), greater PPV (87.0% vs 56.4%, *P* = .01), lower false‐negative rate (20.0% vs 45.1%, *P* = .032), and higher diagnostic accuracy (86.4% vs 68.5%, *P* = .007). Excluding nondiagnostic biopsies (“nondiagnostics”) improved diagnostic metrics for both modalities and preserved the performance advantage of CNB for malignancy, though differences were no longer significant (Table 6).

**Table 5:** Diagnostic Performance for All Parotid Fine Needle Aspiration (FNA) and Core Needle Biopsy (CNB) Biopsies

	Neoplasia		Malignancy		
	FNA (N = 426)	CNB (N = 59)	Statistic	FNA (N = 426)	CNB (N = 59)	Statistic
Sensitivity	85.3%	91.1%	0.3387	54.9%	80.0%	.032*
Specificity	40.0%	66.7%	0.7624	76.2%	91.2%	.077
PPV	92.3%	98.1%	0.22	56.4%	87.0%	.01*
NPV	24.3%	28.6%	1.00	75.1%	86.1%	.209
FPR	60.0%	33.3%	0.76	23.8%	8.8%	.077
FNR	14.7%	8.9%	0.34	45.1%	20.0%	.032*
Accuracy	80.5%	89.8%	0.12	68.5%	86.4%	.007*

Abbreviations: FNR, false negative rate; FPR, false positive rate; NPV, negative predictive value; PPV, positive predictive value.

**Table 6:** Diagnostic Performance for Biopsies Excluding Non‐Diagnostic Results

	Neoplasia			Malignancy		
	FNA (N = 382)	CNB (N = 56)	Statistic	FNA (N = 341)	CNB (N = 56)	Statistic
Sensitivity	93.1%	94.4%	0.945	67.7%	83.3%	.199
Specificity	54.5%	66.7%	1	95.9%	96.9%	1
PPV	95.6%	98.1%	0.639	90.3%	95.2%	.77
NPV	42.9%	40.0%	1	83.9%	88.6%	.639
FPR	45.5%	33.3%	1	4.1%	3.1%	1
FNR	6.9%	5.6%	0.945	32.3%	16.7%	.199
Accuracy	89.8%	93.0%	0.605	85.6%	91.1%	.373

Abbreviations: CNB, core needle biopsy; FNA, fine needle aspiration; FNR, false negative rate; FPR, false positive rate; NPV, negative predictive value; PPV, Positive predictive value.

When evaluating tissue‐specific accuracy, CNB was more accurate than FNA, with 67.8% tissue‐specific accuracy for CNB versus 49.5% for FNA (*P* = .009). This difference persisted with nondiagnostic results excluded (70.2% vs 53.5%, *P* = .018). CNB showed higher tissue‐specific accuracy than FNA at the home institution (*P* < .001) and outside institutions (*P* = .015).

When evaluating factors affecting the accuracy of all biopsies, setting was a significant factor for FNA accuracy for detecting neoplasia (84.6% at the tertiary center vs 75.1% at outside hospitals, *P* = .014). This remained true for diagnosing malignancy, with 74.3% accuracy at the tertiary institution versus 61.1% for FNA at outside hospitals (*P* = .004). CNB accuracy did not depend on the performing site.

For CNB, the number of passes was significant (*P* = .002), with 100% malignancy accuracy when >4 passes were performed (n = 12), and 94% accuracy for ≥3 passes. The gauge used for CNB was a predictor of tissue‐specific diagnostic accuracy, with 100% accuracy using 17 G, 80% using 18 G, and 33.3% using 20 G.

When analyzing only conclusive biopsies, CNB accuracy for malignancy remained associated with the number of passes (*P* = .032) and needle gauge for tissue‐specific diagnosis (*P* = .005). Younger age at biopsy was associated with improved tissue‐specific accuracy (59.0 ± 16.7 years for CNB vs 62.8 ± 15.8 years for FNA, *P* = .017).

We compared the tissue‐specific accuracy of pathologists from outside centers to the tertiary center for biopsies, where pathology was reviewed at both centers. When comparing FNA accuracy from outside pathologists (n = 68) and the home pathologists (n = 58), there was no difference in accuracy (*P* = .629). Similarly, there was no difference in the accuracy of CNB (outside center n = 7 vs tertiary center n = 20, *P* = 1.0).

Prior parotid surgery, prior parotid radiation, BMI, operator specialty, training level, and tumor size were not associated with the accuracy of diagnosing neoplasm, malignancy, or tissue‐specific accuracy.

FNA produced more inconclusive results than CNB (20.0% vs 5.1%, *P* = .004). Biopsy setting influenced frequency of inconclusive diagnoses, with higher rates at outside institutions (24.9%) compared to the home institution (16.2%, *P* = .028). For CNB, a lower number of passes predicted inconclusive results (*P* = .001), with all inconclusive diagnoses occurring when ≤4 passes were performed. Prior parotid surgery, prior parotid radiation, BMI, operator specialty, training level, and tumor size were not associated with inconclusive results.

The rate of unnecessary surgery did not differ significantly between modalities (5.2% for FNA vs 1.7% for CNB, *P* = .24).

When evaluating specific histologic diagnoses, CNB outperformed FNA for squamous cell carcinoma (SCC) and mucoepidermoid carcinoma (MEC). When evaluating all biopsies, 41.7% (n = 20) of FNAs were accurate at diagnosing SCC compared to 100% (n = 6) of CNBs (*P* = .009) (Table 7). For MEC, 14.3% (n = 3) of FNAs were accurate compared to 80.0% (n = 4) of CNBs (*P* = .01) (Table 7). Similar results were observed when nondiagnostics were excluded (Table 8). When evaluating different tumor types, there were no other significant differences between FNA and CNB accuracy for neoplasm, malignancy, tissue‐specific accuracy, inconclusive biopsies, or unneeded surgery.

**Table 7:** Tissue‐Specific Accuracy Including Non‐Diagnostic Results^a^

Surgical pathology result	Biopsy showed accurate tissue‐specific diagnosis	
	FNA (n [%])	CNB (n [%])	*P*‐value
Pleomorphic adenoma	121 (78.6)	15 (88.2)	.53
Warthin tumor	24 (60.0)	7 (70.0)	.72
Squamous cell carcinoma	20 (41.7)	6 (100.0)	.009*
Melanoma	15 (83.3)	2 (100.0)	1.00
Mucoepidermoid carcinoma	3 (14.3)	4 (80.0)	.01*
Acinic cell carcinoma	9 (56.3)	2 (100.0)	1.00
Basal cell adenoma	1 (5.3)	0 (0.0)	.313

Abbreviations: CNB, core needle biopsy; FNA, fine needle aspiration.

^a^ Includes diagnoses with ≥10 total biopsies per final pathologic diagnosis.

**Table 8:** Tissue‐Specific Accuracy Excluding Non‐Diagnostic Results^a^

Surgical pathology result	Biopsy showed accurate tissue‐specific diagnosis	
	FNA (n [%])	CNB (n [%])	*P*‐value
Pleomorphic adenoma	117 (78.5)	15 (88.2)	.53
Warthin tumor	24 (75.0)	7 (77.8)	1.00
Squamous cell carcinoma	19 (42.2)	6 (100.0)	.01*
Melanoma	15 (88.2)	2 (100.0)	1.00
Mucoepidermoid carcinoma	3 (15.8)	4 (80.0)	.01*
Acinic cell carcinoma	9 (60.0)	2 (100.0)	1.00
Basal cell adenoma	1 (7.1)	0 (0.0)	1.00

Abbreviations: CNB, core needle biopsy; FNA, fine needle aspiration.

^a^ Includes diagnoses with ≥10 total biopsies per final pathologic diagnosis.

Among patients who underwent initial FNA at the home institution (n = 231), ROSE was utilized in n = 151 (65.4%) procedures. Otolaryngologists used ROSE least (56%) and radiologists most (96%). ROSE was not associated with lower nondiagnostic rates (odds ratio [OR] 1.1, *P* = .85) or improved tissue‐specific accuracy with neither nondiagnostics included (OR 0.99, *P* = .97) nor excluded (OR 1.0, *P* = .99). Among nondiagnostics, 46% had ROSE results showing insufficient sampling, and 54% demonstrated atypical cells without definitive diagnosis.

## Discussion

In this retrospective study of 485 patients who underwent parotidectomy, we found that CNB offers superior diagnostic accuracy, particularly for malignant tumors and tissue‐specific diagnoses such as SCC and MEC, without increasing complications or delaying care. While FNA remains a valuable, less invasive tool, CNB may be considered in cases with high clinical or radiographic suspicion for malignancy, particularly when preoperative histologic specificity would alter surgical planning.

The FNA sensitivity reported in the literature is highly variable (59%‐93.5%, mean 74.1%), with specificity averaging 93.2%.^8^ We are among the first to report diagnostic performance for both neoplasm and malignancy. Our results suggest FNA sensitivity for malignancy (54.9%) sits at the lowest end of published estimates and highlights the likely overestimation of FNA performance by many studies. Our FNA nondiagnostic rate of 20.0% is more than double the 8.1% reported in prior systematic reviews.^9^ This likely reflects inclusion of biopsies from outside institutions with potentially less experienced technique or lack of on‐site cytopathology, as supported by higher inconclusive biopsy rates from outside institutions.

Despite prior studies demonstrating the utility of ROSE for FNA, such as one report of 60 salivary gland FNAs and another of 248 parotid FNAs showing 100% adequacy and excellent agreement with final pathology, respectively,^10^, ^11^ our institutional experience did not reveal a difference in rates of inconclusive biopsies or accuracy compared to final pathology when ROSE was utilized for FNAs. The high inconclusive sample rate, with half sent for formal review despite inadequacy, suggests either technical issues with sample acquisition (eg, due to tumor size/location) or ROSE application. Limited ROSE use (56% of FNAs) by otolaryngologists may introduce selection bias for complex tumors, making our ROSE findings less generalizable.

There were no differences in pathologist accuracy between community sites and the tertiary center, suggesting biopsy technique is most critical and not the pathologist. This underscores the high dependency of FNA cytology on operator skill and sampling adequacy. Across home and outside settings, we observed that radiologists performed CNB significantly more often than otolaryngologists, suggesting that biopsy modality selection may be influenced by performing specialty.

The CNB nondiagnostic rate was 5.1% in our study, comparable to the 1.2% reported in the literature.^12^ Performance of CNB is frequently reported as higher and more consistent than FNA (sensitivity 81.1%‐96.7%, specificity 85.7%‐100%).^3^, ^4^, ^8^, ^9^, ^13^, ^14^ Our results are similar but emphasize improved diagnostic performance for malignancy across all metrics. The lower nondiagnostic rate with CNB may reduce need for repeat biopsies, which decreases anxiety, delays in diagnosis, and costs. CNB may reduce performance variability across settings and improve tissue‐specific diagnosis because it preserves architecture and enables ancillary testing (eg, immunohistochemistry/flow cytometry).

CNB outperformed FNA in tissue‐specific analysis, helping providers educate patients on the diagnosis preoperatively. This was especially true for malignancy, where FNA correctly identified only 14.3% of MEC cases and 41.7% of SCC cases, compared to 100% and 80% by CNB. Low‐grade MEC is frequently underdiagnosed due to its bland, mucin‐rich cytology, which often mimics benign ductal or cystic lesions such as pleomorphic adenoma.^15^, ^16^ SCC can also be difficult to diagnose as it can mimic salivary malignancies, atypical cells can look like reactive metaplasia, and cystic components can lead to inconclusive results.^17^ Our cohort had a high proportion of malignant cutaneous metastases, reflecting real‐world biopsy selection for high‐pretest probability lesions. While rapid growth, pain, functional symptoms, immunosuppression, and concerning imaging likely influenced decisions, such factors were not systematically collected nor analyzed. Our findings suggest CNB may be useful for lesions suspicious for malignancy or where tissue‐specific diagnosis is critical for operative planning. Our study cannot directly prove CNB changes downstream management, and our recommendations are guidance, not definitive evidence.

Two modifiable techniques for CNB emerged. First, accuracy improved when ≥3 passes were performed, with 100% accuracy observed with ≥4 passes obtained. Second, a larger needle gauge improved tissue‐specific diagnosis, with 100% accuracy using 17 G needles and decreasing accuracy with smaller gauges. These findings provide targets for procedural optimization, recommending ≥3 passes with ≥18 G needles. By contrast, operator specialty, training level, and patient characteristics such as BMI and tumor size were not associated with accuracy, suggesting that how much tissue is obtained may matter more than who obtains it or who it is obtained from.

Both FNA and CNB were safe, with low and comparable complication rates and no observed CN7 injuries or tumor seeding events. Our findings are similar with prior pooled analysis of 1315 CNBs that recorded only one transient palsy attributable to anesthetic.^14^ We observed no cases of needle‐track seeding. Evidence of this complication is limited to two case reports from the 1990s,^18^, ^19^ and contemporary authors agree that fear of seeding should not influence biopsy choice.^20^ Turnaround times for pathology review indicate no delay in the diagnostic workflow of CNB. While complication rates were low, and no tumor seeding events were observed, we acknowledge that the study is not powered to detect rare complications, and longer follow‐up is needed to compare uncommon adverse outcomes.

Despite CNB's advantages, FNA may retain a role for cases of low pretest probability of malignancy in centers with immediate on‐site cytopathology, patients who cannot tolerate larger‐gauge needles, faster biopsy after a low‐risk imaging and benign examination, or resource‐limited settings where CNB cost, expertise, or pathology infrastructure for processing is not available. In many institutions, a tiered strategy—including FNA first with immediate assessment, escalating to CNB for nondiagnostic or suspicious results—remains pragmatic. Our data, however, suggest that in lesions with high suspicion for malignancy, or where a histology‐level diagnosis would significantly change management, CNB should be considered. Cost effectiveness, availability, and patient experience should be further studied.

This study was not designed to assess the impact of biopsy modality on downstream clinical decision‐making. By restricting inclusion to patients who underwent parotidectomy, patients with true‐negative biopsies who did not proceed to surgery were excluded, introducing spectrum bias and limiting inference regarding management changes, such as observation versus surgery. Consequently, results may not be generalizable to all parotid masses, particularly those managed with observation. Future studies incorporating nonoperative cohorts should evaluate how biopsy modality influences treatment selection.

Several limitations warrant consideration. Retrospective design limits control over variables such as operator experience, biopsy technique, and pathology interpretation. Clinicians may have preferentially selected CNB for lesions with a higher pretest probability of malignancy, which may limit generalizability and affect predictive values. The relatively small number of CNBs (n = 59) reduces precision in subgroup analyses and limits the ability to draw strong conclusions for less common histologies. Because this was a retrospective parotidectomy‐only cohort with univariate analyses and incomplete capture of biopsy‐selection factors, any apparent CNB advantage should be viewed as associative rather than causal or generalizable. Multivariable regression was not performed due to the limited number of CNBs and malignant outcomes and collinearity between biopsy modality, operator specialty, and biopsy setting, which precluded construction of a stable and interpretable model and may limit adjustment for confounding. Follow‐up data outside our health system were limited. Finally, this was a single‐center study conducted at a high‐volume tertiary care institution with subspecialty expertise, which may limit generalizability to lower‐volume centers, though we attempted to overcome this by including biopsies performed at outside community institutions.

## Conclusion

In this cohort, CNB was associated with higher diagnostic accuracy than FNA, particularly for malignant tumors and tissue‐specific diagnoses, without increasing complications or delaying care. Standardizing the CNB technique to ≥3 passes with 17 to 18 G needles may enhance diagnostic accuracy and reduce inconclusive results. Future work should include prospective, multicenter comparisons standardizing on‐site cytopathology, cost‐effectiveness analyses weighing higher upfront resource utilization of CNB against fewer repeat procedures, and development of decision support algorithms integrating clinical, radiographic, and institutional factors to guide the optimal first biopsy choice.

## Author Contributions

**Lisa Eleni Chionis Buhler** and **Andrew Prince**: conceptualization, data curation, formal analysis, investigation, methodology, project administration, supervision, validation, visualization, writing—original draft, writing—review and editing. **Nicholas Bunda**: formal analysis, visualization, writing—original draft, writing—review and editing. **Kimberly Oslin**, **Michael Allevato**, and **Emma Hershey**: investigation, data curation, writing—review and editing. **Joshua Smith**: conceptualization, supervision, writing—review and editing. **Steven B. Chinn** and **Mark E. P. Prince**: conceptualization, supervision, writing—review and editing.

## Disclosures

### Competing interests

The authors declare no conflicts of interest.

### Funding source

None.
